# Sialylation inhibition improves macrophage mediated tumor cell phagocytosis of breast cancer cells triggered by therapeutic antibodies of different isotypes

**DOI:** 10.3389/fonc.2024.1488668

**Published:** 2024-11-26

**Authors:** Marta Lustig, Christoph Hahn, Marie Leangen Herigstad, Jan Terje Andersen, Jeanette H. W. Leusen, Renate Burger, Thomas Valerius

**Affiliations:** ^1^ Division of Stem Cell Transplantation and Cellular Immunotherapies, Department of Medicine II, University Medical Center Schleswig-Holstein and Christian-Albrechts-University Kiel, Kiel, Germany; ^2^ Institute for Clinical Medicine, Department of Pharmacology, University of Oslo and Oslo University Hospital, Oslo, Norway; ^3^ Institute for Clinical Medicine, Department of Immunology, University of Oslo and Oslo University Hospital, Oslo, Norway; ^4^ Precision Immunotherapy Alliance (PRIMA), University of Oslo, Oslo, Norway; ^5^ Center for Translational Immunology, University Medical Center Utrecht, Utrecht, Netherlands

**Keywords:** immune checkpoint, sialic acid, Siglecs, myeloid cells, therapeutic antibodies, IgG, IgA

## Abstract

Tumor cell phagocytosis by macrophages is considered a relevant mechanism of action for many therapeutic IgG antibodies. However, tumor cells employ several mechanisms to evade immune recognition, including hypersialylation. Here, we describe how reduction of sialic acid exposure on tumor cells promotes antibody-dependent tumor cell phagocytosis (ADCP) by macrophages. Incubation with the sialyltransferase inhibitor (STi) P-3Fax-Neu5Ac reduced sialylation on two breast cancer cell lines, rendering these cells more susceptible to macrophage mediated phagocytosis by EGFR or HER2 antibodies. This was observed with not only IgG1 and IgG2 antibodies but also IgA2 variants. These results show that inhibiting sialic acid exposure triggers enhanced tumor cell phagocytosis by macrophages irrespective of the antibody isotype and the tumor target antigen. Investigating the underlying mechanisms of enhanced ADCP, we observed reduced binding of soluble sialic acid-binding immunoglobulin-like lectins (Siglec)-7 and Siglec-9 to tumor cells after sialylation inhibition. However, Fc silent blocking antibodies against Siglec-7 or Siglec-9, or their combination, only marginally improved ADCP. Our results further promote the concept of cancer hypersialylation as immune escape mechanism, which could serve as target to improve tumor immunotherapy with monoclonal antibodies.

## Introduction

Antibodies against immune checkpoint molecules on T cells – such as CTLA-4 or PD-1 - have revolutionized cancer immunotherapy – leading to the Nobel Prize in Medicine being awarded to J. P. Allison and T. Honjo in 2018 (1). Meanwhile, monoclonal antibodies interfering with PD-1/PD-L1 or CTLA-4/CD28 interactions have also been approved by the FDA (2). These inhibitors are used for the treatment of almost half of all cancer patients in the United States, however, more than 80% of these patients remain unresponsive to or progress during this type of immune checkpoint therapy (3).

Myeloid cells constitute a major cell population in the tumor (4), but are mainly suppressive immune cells, and their presence is often associated with cancer progression (5, 6). On the other hand, myeloid cells, and macrophages in particular, have been reported to be the predominant effector cell population for antibodies against tumor associated antigens such as HER2, EGF-R or CD20 (7, 8). For this reason, targeting myeloid cells by reversing their immunosuppressive features may be a promising approach. Indeed, in antibody-based cancer therapy macrophages can be activated to kill tumor cells by antibody-dependent cellular phagocytosis (ADCP) via activating Fcγ receptors (9). Additionally, macrophages are important target cells of novel checkpoint blockade approaches (10).

The first and very promising immune checkpoint identified on myeloid cells is the inhibitory receptor signal regulatory protein alpha (SIRPα), which interacts with integrin associated protein (IAP; CD47) on cancer cells (11, 12). The interaction between CD47 and SIRPα initiates inhibitory downstream signaling in myeloid cells. Physiologically, CD47 functions as “don´t eat me” signal to restrict phagocytic function (11, 12). CD47 is known to be upregulated in patients with different cancer types and is considered a poor prognostic marker (13, 14). Indeed, cancer cells hijack the CD47-SIRPα interaction by upregulating CD47 (13, 14) and blocking CD47 on cancer cells has been demonstrated to improve the efficacy of different therapeutic antibodies in inducing ADCP in several preclinical mouse models (15, 16). The CD47 blocking IgG4 antibody magrolimab is the first myeloid checkpoint inhibitor that has been tested in clinical trials. After promising results in combination with rituximab in non-Hodgkin’s lymphoma patients (17), recent clinical trials of magrolimab were placed on hold due to inefficacy compared to standard therapies and increased risk of death (18). Nevertheless, magrolimab has confirmed the scientific rationale to target myeloid checkpoints in cancer, opening new therapeutic perspectives.

Altered glycosylation is common in cancer and among various glycosylation changes, cancer cells from different origins increase their cell surface sialoglycans, favoring cancer growth, progression and immune escape (19–21). The underlying cause of surface hypersialylation of cancer cells is the upregulation of sialyltransferases or the alteration of neuraminidase expression, which correlates with poor prognosis (19, 21). Furthermore, aberrant sialylation contributes to immune evasion through the family of sialic acid-binding immunoglobulin-like lectins (Siglecs) that includes inhibitory receptors expressed by immune cells (22). There is sufficient evidence that the sialic acid-Siglec interaction is an important immune checkpoint for NK and T cells, but the knowledge about its involvement in myeloid cell mediated tumor cell lysis is still limited, especially in the context of antibody mediated effector functions (23, 24).

Myeloid cells express high levels of Siglec-3 (CD33), Siglec-7 and -9 which regulate their life span in inflammatory responses to avoid tissue damage (21, 25). Furthermore, blockade of CD24 interactions with Siglec-10, expressed by tumor-associated macrophages, has been shown to cause enhanced phagocytic activity against solid cancer cells (26). Recently, sialoglycans have been shown to function in trans and cis as “don’t eat me” and “don’t eat” signals engaging Siglecs on macrophages (27). In addition, targeting human Siglec-7 and -9 on myeloid cells of transgenic mice has shown to be a promising strategy in cancer-targeting immunotherapy (28). The shared expression of inhibitory Siglecs on different immune cells and the multiple roles of sialic acid mentioned above, make the sialic acid/Siglec axis an attractive immune checkpoint to target for therapeutic purposes.

This study investigates the role of sialic acid in the regulation of ADCP by macrophages using breast cancer cell lines. As a strategy to reduce sialylation on cancer cells, a sialyltransferase inhibitor was used to address its effect on tumor cell phagocytosis. The results showed that reduction of sialic acid on tumor cells promoted ADCP by macrophages by the use of EFGR and HER2 antibodies of IgG1 or IgG2 isotypes as well as recombinant IgA2 variants with the same tumor antigen specificities. Taken together, we demonstrate that sialic acid inhibits antibody-dependent phagocytic activity of myeloid cells and that this is a promising target that opens new perspectives in cancer immunotherapy.

## Materials and methods

### Cell lines and culture

The human breast cancer cell lines MDA-MB-468 and SK-BR-3 were used as target cells for EGFR and HER2 antibodies, respectively. Both cell lines were obtained from the German Collection of Microorganisms and Cell Cultures (DSMZ, Braunschweig, D) and cultured in RPMI 1640 media supplemented with 10% heat-inactivated fetal calf serum and antibiotics in a humidified 5% CO_2_ atmosphere at 37°C. Cells were regularly monitored for *Mycoplasma* infection using PCR-based methods.

### Antibodies

The clinically approved antibodies cetuximab (chimeric human IgG1; clone 225, Erbitux^®^) and panitumumab (human IgG2; clone E7.6.3, Vectibix^®^), both binding the EGFR, were from Merck (Darmstadt, DE) and Amgen (Thousand Oaks, CA, USA). Trastuzumab (humanized IgG1; clone 4D5-8; Herceptin^®^) against HER2 was from Roche (Basel, CH), while a recombinant IgG2 form of trastuzumab was produced as described (29). Recombinant IgA2.0 variants based on the variable sequences derived from cetuximab and trastuzumab were generated as described (30, 31). Siglec-7 (1E8) and Siglec-9 (mAbA) blocking antibodies were produced as Fc silent variants (based on mouse IgG1 or human IgG2, respectively) using published sequences (28, 29, 32, 33).

### Inhibition of cell surface sialylation

Sialylation on the surface of cancer cells was inhibited pharmacologically using the fluorinated sialic acid analogue P-3Fax-Neu5Ac as a sialyltransferase inhibitor (STi) (MerckMillipore, Burlington, MA, USA) (34). Cells were incubated with the STi dissolved in DMSO at a final concentration of 100 µM for 72 h at 37°C.

### Flow cytometry

Immunofluorescence analyses were performed on a Navios flow cytometer and the Kaluza software (Beckman Coulter). For characterization of human macrophages, purified mouse monoclonal antibodies directed against CD14, CD86, CD163, CD206 (BioLegend, San Diego, CA, USA) and CD68 (eBioscience™/ThermoFisher Scientific) were used. Binding of mouse antibodies was detected using fluoresceinisothiocyanat (FITC)-conjugated goat anti-mouse Fcγ-specific F(ab’)_2_ fragments (30 min, 4°C) (Jackson ImmunoResearch Laboratories, West Grove, PA, USA). Expression levels of Fc receptors and Siglecs on macrophages were quantified by determination of specific antigen binding capacities (SABC) of monoclonal mouse antibodies (all from BioLegend) (50 µg/ml, 1 h, 4°C) using the QIFIKIT^®^ (Agilent/DAKO, Glostrup, Denmark). EGFR and HER2 receptors on cancer cells were detected using cetuximab and trastuzumab (10 µg/ml, 30 min, 4°C), irrelevant human IgG1 was used as control. Recombinant soluble human Siglec-Fc chimeric proteins (R&D Systems, Minneapolis, MN, USA) were used to assess the presence of Siglec-binding epitopes on tumor cells (10 µg/ml for 1 h at 4°C). Binding of human IgG antibodies and Siglec-Fc fusion proteins was detected using phycoerythrin (PE)-conjugated goat anti-human Fcγ-specific F(ab’)_2_ fragments (30 min, 4°C) (Jackson ImmunoResearch Laboratories). Sialic acid expression on cancer cells was assessed by binding of biotinylated Maackia amurensis leukagglutinin (MAL) II (5 µg/ml) detected with streptavidin-PE. Siglec-7 mIgG1 and Siglec-9 hIgG2 antibodies were detected with FITC-conjugated goat anti-mouse and anti-human Fcγ-specific F(ab)_2_ fragments, respectively.

### Generation of macrophages

Monocytes were isolated from peripheral blood of healthy volunteers after informed consent according to the ethical approval of the University of Kiel. Briefly, peripheral blood mononuclear cells were cultured in monocyte attachment medium (PromoCell, Heidelberg, D) for 30 min at 37°C, and non-adherent cells were removed. Adherent monocytes were differentiated into macrophages by culturing in serum-free X-vivo medium (Lonza, Basel, CH) supplemented with 50 ng/ml recombinant macrophage colony-stimulating factor (M-CSF; PeproTech, Hamburg, D) for 11 to 14 days and then used for phagocytosis assays.

### ADCP

For use in ADCP, differentiated macrophages were detached from the plate by incubation in dissociation buffer (ThermoFisher Scientific) at room temperature (RT) for 20 min, seeded into a 96-well flat-bottom plate, and allowed to adhere at RT for 30 min. Antibodies were added at a final concentration of 10 μg/ml. Cancer cells were labelled with the pH-sensitive red fluorescent dye pHrodo (ThermoFisher Scientific) (5 µg/ml in PBS at RT in the dark for 60 min) and added to the plate at an effector to target cell (E:T) ratio of 1:1 (20,000:20,000 cells). All samples were performed in triplicate. The plate was incubated at 37°C and subjected to live cell fluorescence imaging using the IncuCyte^®^ high-throughput fluorescence microscope system (Sartorius). Four fluorescence pictures (magnification 20 x) of each well were created every 30 minutes up until 10 h. Phagocytosis was determined as the red object counts per image (ROI), representing the phagocytosed cancer cells, over time.

### SDS-PAGE

Purified Siglec antibodies were separated by SDS-PAGE under non-reducing and reducing conditions using a 4-15% precast ^®^TM polyacrylamide gel (Mini-PROTEAN TGX, BioRad). After a running time of 90 min at constant 120 V, gels were stained with Coomassie brilliant blue staining solution (Carl Roth, Karlsruhe, DE).

### Statistical analysis

At least three independent experiments were performed for flow cytometric analyses and in ADCP assays with effector cells from different donors (triplicates for each). Data were statistically analyzed with GraphPad Prism Software 10 (San Diego, CA, USA). Significance was accepted with p ≤ 0.05.

## Results

### Characterization of macrophages

**Figure 1:**
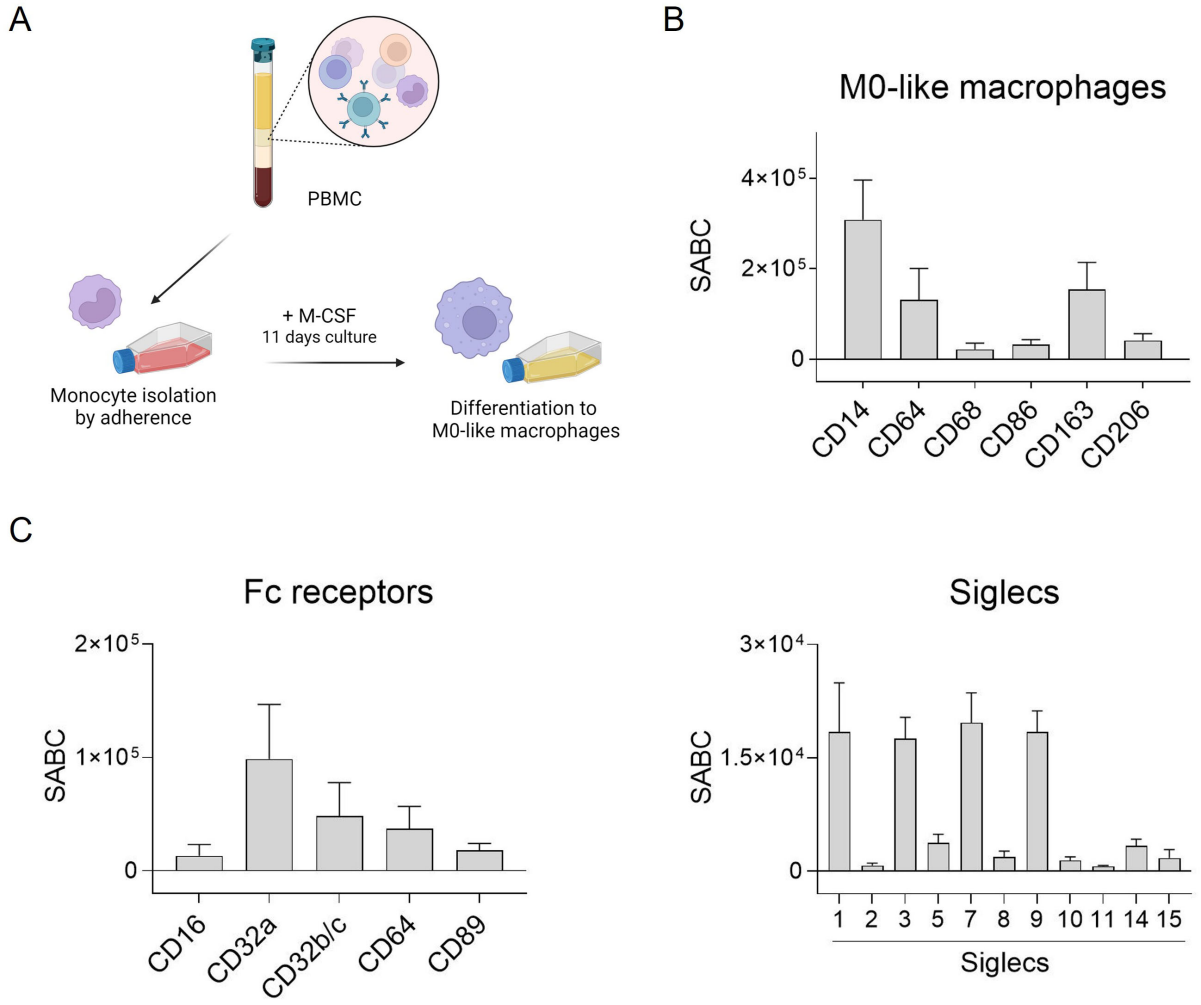
Fc receptor and Siglec expression by human M0-like macrophages. **(A)** Schematic representation of M0-like macrophage differentiation from peripheral blood mononuclear cells (PBMC). **(B, C)** Expression of M0-like markers, Fc receptors and Siglecs was analyzed by flow cytometry. Cells were incubated with specific mouse monoclonal antibodies (50 µg/ml) and binding was detected with FITC-conjugated goat anti-mouse Fcγ-specific F(ab)_2_ fragments. Quantitative antigen expression levels as determined with the QIFI kit (Dako, Glostrup, Denmark) are depicted as mean specific antibody binding capacities (SABC) ± SEM of 3 independent replicates with differentiated macrophages from different donors.

Several studies have highlighted the crucial role of macrophages in antibody-based immunotherapy (35). To further investigate this, we generated macrophages from peripheral human blood monocytes to assess the effectiveness of different antibody isotypes in activating tumor cell phagocytosis. Monocyte-derived macrophages were produced through adhesion, and stimulated with M-CSF to induce M0-like differentiation (**Figure 1A**). Subsequently, typical macrophage markers were assessed using direct immunofluorescence. The macrophages exhibited high CD14, CD64 and also CD163 expression, while other markers like CD68, CD86 and CD206 were expressed at lower levels (**Figure 1B**).

Antibody-dependent tumor cell killing by macrophages is mediated by binding of antibodies to Fc receptors (FcR) which can transmit activating signals into immune effector cells. Thus, expression of FcR on macrophages was quantitatively analyzed by flow cytometry. The most abundantly expressed FcR on macrophages generated from peripheral blood monocytes was FcγRIIa (CD32a), followed by FcγRIIb/c (CD32b/c), FcγRI (CD64), and FcαRI (CD89) (**Figure 1C**, left panel). The quantitative expression levels of Siglecs was analyzed similarly. Some of the human Siglecs were excluded, e.g. Siglec-4, as it is not expressed on leukocytes, Siglec-6 (although expressed on basophils, subsets of B cells and dendritic cells (36, 37)), Siglec-12 which has lost sialic acid binding capacity, and Siglec-13 which is deleted in humans. Among the eleven Siglecs analyzed, our macrophages were shown to express Siglec-1, -3, -7, and -9 (**Figure 1C**, right panel).

### Effect of a sialyltransferase inhibitor on cancer cell sialylation

**Figure 2:**
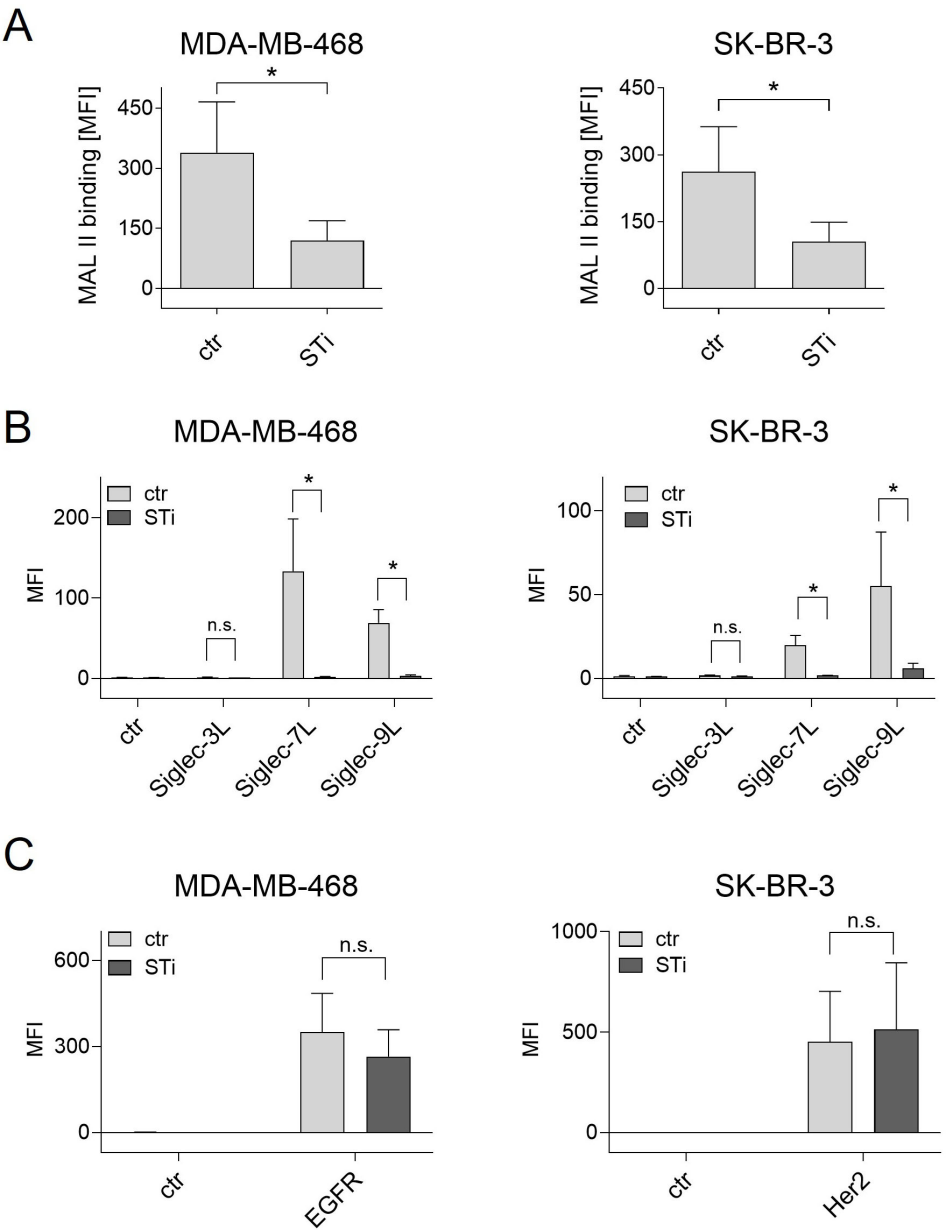
Treatment of cancer cells with a sialyl transferase inhibitor (STi) reduced sialylation and the presence of Siglec ligands on the cell surface. **(A)** Binding of biotinylated Maackia amurensis leukagglutinin (MAL) II (5 µg/ml) on MDA-MB-468 and SK-BR-3 cells was detected with streptavidin-PE. PBS was used with streptavidin-PE as control. **(B)** Binding of soluble Siglec-3/-7/-9-Fc proteins (10 µg/ml) on untreated or STi treated breast cancer cells (P-3Fax-Neu5Ac, 100 µM, 72 h, 37°C) was detected with PE-conjugated goat anti-human Fcγ-specific F(ab)_2_ fragments. An irrelevant human IgG1 antibody was used as control (ctr). **(C)** Treatment of cancer cells with the STi did not significantly affect the binding of cetuximab or trastuzumab (each 10 µg/ml) to EGFR or HER2, respectively. Cetuximab and trastuzumab binding was detected with PE-conjugated goat anti-human Fcγ-specific F(ab)_2_ fragments. Shown are the mean MFI values ± SEM of 3 independent experiments. Statistical differences between treated and untreated cells were analyzed with one-way ANOVA. (*) p ≤ 0.05. SiglecL, Siglec ligand; STi, sialyltransferase inhibitor; MFI, mean fluorescence intensity; n.s., not significant.

The effect of a sialyltransferase inhibitor on cancer cell sialylation and Siglec ligand exposure was assessed by flow cytometric binding studies. First, binding of MAL II to sialic acid on breast cancer cell lines was reduced after STi treatment (**Figure 2A**). Second, soluble Fc fusion proteins of the three Siglecs that are highly expressed on macrophages, namely Siglec-3, -7, and Siglec-9, were analyzed for their capacity to recognize Siglec ligands on MDA-MB-468 or SK-BR-3 cells. Siglec-1 ligands were not analyzed, since Siglec-1 does not have an intracellular inhibitory domain (22). Both cell lines showed significant binding of Siglec-7-Fc and Siglec-9-Fc, while binding of Siglec-3-Fc was only marginal or not detectable (**Figure 2B**). The mean MFI value for Siglec-7-Fc binding on MDA-MB-468 cells was 133 ± 65, on SK-BR-3 the MFI value was much lower. Binding of Siglec-9-Fc had a mean MFI value of 69 ± 16 on MDA-MB-468 cells and 55 ± 32 on SK-BR-3 cells. Importantly, treatment of MDA-MB-468 and SK-BR-3 cells with the STi P-3Fax-Neu5Ac resulted in almost complete abrogation of Siglec binding (**Figure 2B**). In contrast, the reduction of cell surface sialic acid by treating cells with STi had no effect on EGFR or HER2 expression (**Figure 2C**). Thus, it can be concluded that a feasible approach to reduce sialic acid exposure on tumor cells, and thereby Siglec binding to macrophages, is the application of a sialyltransferase inhibitor, at least *in vitro*.

### Inhibition of sialylation improves ADCP

**Figure 3:**
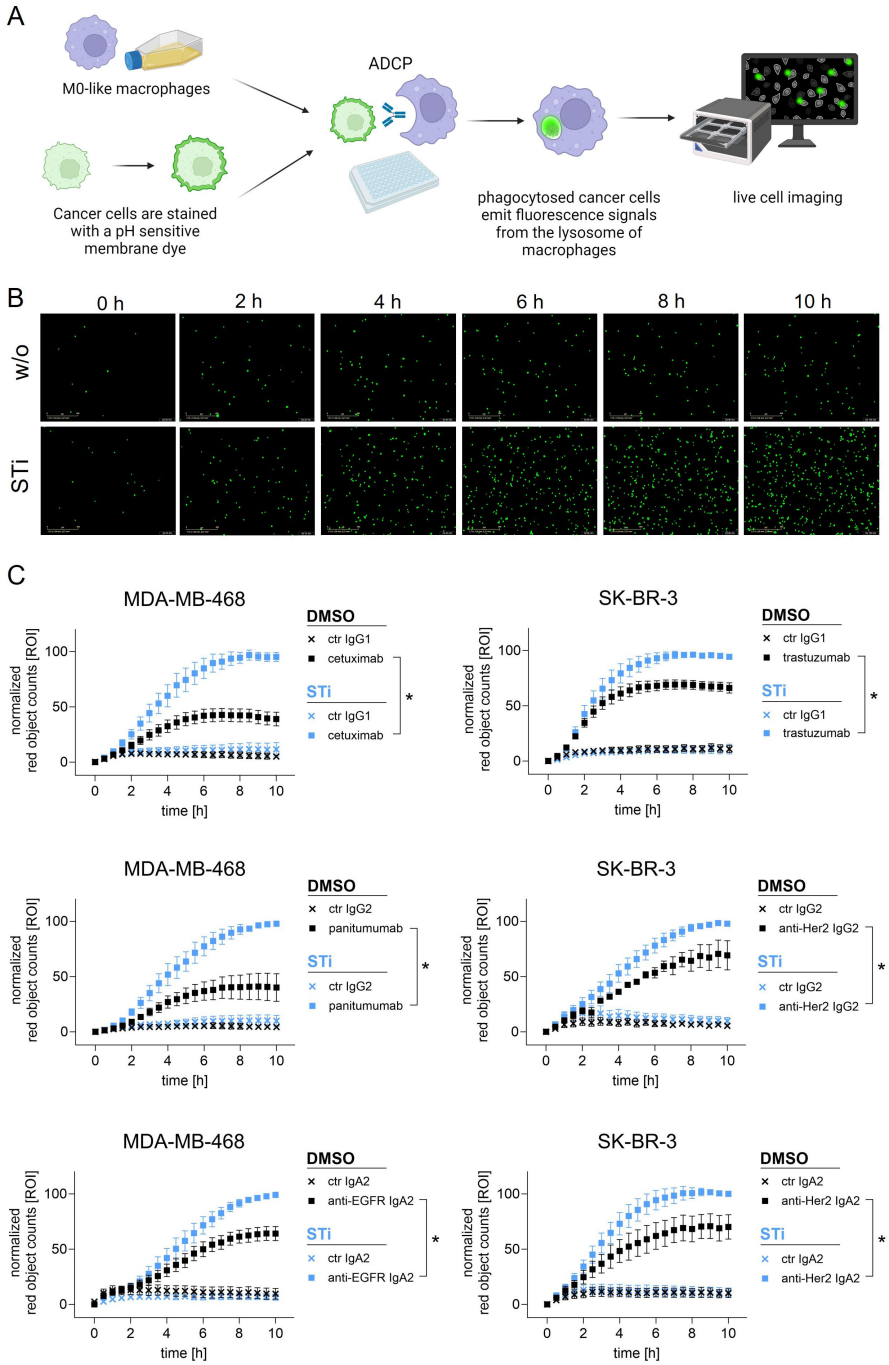
Sialylation inhibition on cancer cells by treatment with STi improved ADCP by macrophages. **(A)** Schematic representation of ADCP measured by live cell imaging. **(B)** Microscopic images depicting phagocytosed MDA-MB-468 cells (in green) in the presence of panitumumab (10 µg/ml) over time (0 h to 10 h). Magnification 20 x. **(C)** Phagocytosis rates of MDA-MB-468 and SK-BR-3 cells in the presence of antibodies over time. MDA-MB-468 and SK-BR-3 cells were treated with STi (P-3Fax-Neu5Ac, 100 µM, 72 h, 37°C) or DMSO as control. Cetuximab and panitumumab were used to target EGFR, and trastuzumab or anti-HER2 IgG2 to target HER2. IgA2 antibodies carrying the variable regions of cetuximab or trastuzumab were used to target EGFR or HER2, respectively. All antibodies were used at 10 µg/ml. Human macrophages were applied at an effector to target cell (E:T) ratio of 1:1. Results were normalized to the highest value (red objects per image, ROI) of phagocytosed cells and shown as the mean percentage ± SEM of at least n=3 independent experiments with macrophages from different donors, each performed in triplicates. Data were analyzed by two-way ANOVA. (*) depicts significant differences in the curve trend (p ≤ 0.05) between STi treated and DMSO control cells.

**Figure 4:**
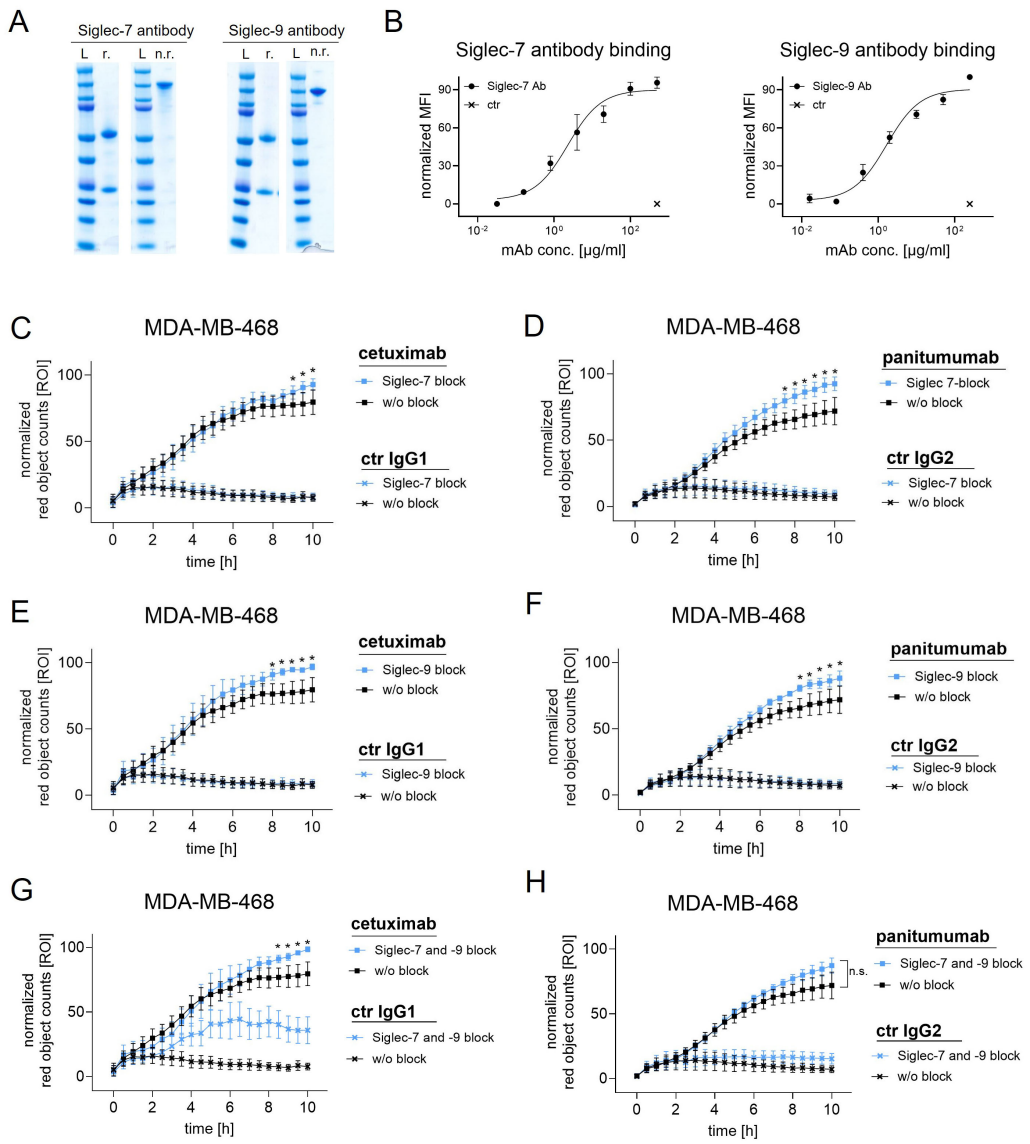
Siglec-7 and Siglec-9 blocking antibodies with a silent Fc part only slightly improved ADCP by macrophages. **(A)** SDS-PAGE of Siglec-7 (mIgG1) and Siglec-9 (hIgG2) blocking antibodies. r., under reducing conditions; n.r., non-reducing conditions. L, ladder. **(B)** Binding curves of Siglec-7 (500, 100, 20, 4, 0.8, 0.16, 0.032 µg/ml) and Siglec-9 (250, 50, 10, 2, 0.4, 0.08, 0.016 µg/ml) blocking antibodies on macrophages. Siglec-7 and Siglec-9 antibodies were detected with FITC-conjugated Fcγ-specific F(ab)_2_ fragments (used alone as negative control). Ab, antibody. **(C-H)** Phagocytosis rates of MDA-MB-468 cells in the presence of cetuximab or panitumumab (both at 10 µg/ml) in combination with Siglec-7 (100 µg/ml) or Siglec-9 (20 µg/ml) blocking antibodies, or the combination of both, over time. Human macrophages were applied at an effector to target cell (E:T) ratio of 1:1. Results were normalized to the highest value (red objects per image, ROI) of phagocytosed cells and shown as the mean percentage ± SEM of at least 3 independent experiments with macrophages from different donors, each performed in triplicates. Data were analyzed by two-way ANOVA. (*) depicts significant differences (p ≤ 0.05) between therapeutic antibodies alone or in combination with Siglec blocking antibodies.

To investigate the role of sialylation, MDA-MB-468 and SK-BR-3 breast cancer cells were treated with STi and used as target cells for macrophages in the presence of therapeutic monoclonal antibodies (**Figure 3A**). As shown in microscopic images, the number of phagocytosed MDA-MB-468 cells in the presence of panitumumab (anti-EGFR human IgG2) increased over time and was much higher with STi treated cells versus DMSO treated control cells (**Figure 3B**). This was also demonstrated for cetuximab (anti-EGFR, human IgG1), trastuzumab (anti-HER2/neu, humanized IgG1) and a recombinant IgG2 variant of trastuzumab. Phagocytosis rates over time are shown in **Figure 3C**. The curves reveal that the capacity of macrophages to phagocytose cancer cells in the presence of antibodies was significantly enhanced when cells were treated with STi compared to DMSO treated control cells. Moreover, the presence or absence of sialic acid has an influence on cancer cell phagocytosis triggered by IgA antibodies, as shown for IgA2 variants based on the variable regions of cetuximab or trastuzumub, respectively (**Figure 3C**). For both variants, ADCP was increased when target cells were treated with STi compared to DMSO treated control cells. To investigate whether the improvement of ADCP provided by STi is caused by reduced binding to the relevant Siglecs expressed on macrophages, Siglec-7 or Siglec-9 were blocked using Fc silent antibodies (**Figure 4A**). As shown in **Figure 4B**, both antibodies showed concentration-dependent binding to macrophages. However, applying these antibodies in functional ADCP assays (**Figures 4C–H**), macrophage driven ADCP in the presence of cetuximab or panitumumab was only slightly enhanced and much less efficient than after sialyltransferase inhibition (**Figure 3**). Taken together, these data show that the reduction of cell surface sialic acid levels can increase ADCP of cancer cells, irrespective of the antibody isotype - IgG1, IgG2, or IgA2. However, sialic acid/Siglec interactions, at least with regard to Siglec-7 or Siglec-9, appeared not to be responsible for attenuating myeloid cell mediated phagocytosis.

## Discussion

Myeloid cells are commonly present in the microenvironment of many solid cancers, and myeloid cell infiltrates are often associated with immunosuppressive functions and a poor prognosis (5, 6). On the other hand, recent studies in immunodeficient NSG mice with only a functional myeloid, but no lymphoid compartment, demonstrated therapeutic antibody efficacy - further confirming the therapeutic rational of recruiting myeloid cells in antibody therapy (15, 38). Indeed, macrophages have long been reported to be an important effector cell population for many monoclonal antibodies, including cetuximab and trastuzumab (35). Nevertheless, similar to T cells, myeloid cell activation is suppressed by immune checkpoint molecules.

The sialic acid- Siglec axis is gaining attention as novel potential immune checkpoint for different immune cells, including myeloid cells. Indeed, in cancer progression, cancer cells undergo hypersialylation to evade immunosurveillance. For instance, cancer cell hypersialylation suppresses the immune system by engaging inhibitory Siglecs on immune cells. In macrophage driven phagocytosis of cancer cells, Siglec-7, -9, and -10 have already been described to be the most important Siglecs with various modulatory effects (39). in this study, macrophages mainly expressed Siglec-1, -3, -7 and Siglec-9, while Siglec-10 was not expressed by our M0-like macrophages (26). Except for Siglec-1, which does not have an intracellular ITIM domain (22), the inhibitory Siglecs found to be expressed by our differentiated M0-like macrophages, were tested for binding as soluble Fc fusion proteins to the cancer cell lines used. The employed breast cancer cell lines expressed ligands for Siglec-7 and Siglec-9, but not for Siglec-3. Importantly, binding of Siglec-7- and -9-Fc proteins was reduced when the cancer cells were treated with STi. In general, ligands for Siglec-7 and -9 are often increased in several cancers, which may be a hint that cancer cells undergo hypersialylation to escape myeloid cell surveillance in a Siglec dependent manner (24, 40). In pancreatic ductal adenocarcinoma e.g., overexpression of α2,3 linked sialic acid contributes to the differentiation of monocytes to immune suppressive tumor associated macrophages (TAM) by engaging Siglec-9 and Siglec-7, while Siglec-9 is responsible for production of the anti-inflammatory cytokine interleukin (IL)-10 by macrophages (40, 41). Here, sialic acid removal by STi also enhanced human IgG1 and IgG2 mediated ADCP of HER2 and EGFR positive breast tumor cells. Additionally, myeloid cells express FcαRI and can be activated by preclinical IgA2 antibodies to perform phagocytosis of cancer cells (42, 43). Inhibition of sialyation on cancer cells also enhanced ADCP mediated by IgA2 antibodies. Similar to neutrophils (29, 44), the effector function of macrophages, driven by different antibody isotypes, is also regulated by sialic acid.

In this study, we observed that Fc silent blocking antibodies targeting Siglec-7 or Siglec-9, individually or in combination, only modestly enhanced ADCP by macrophages when using either panitumumab (human IgG2) or cetuximab (human IgG1). This effect was not as substantial as that achieved with sialyltransferase inhibition, suggesting a limited role for Siglecs in attenuating ADCP compared to sialylation inhibition. Prior research indicated that recruitment of Siglecs to Fc receptors (FcR) can fully suppress FcR activation by antibodies (45). In this case, the spatial organization between FcR and Siglecs, particularly within the immunological synapse, appears critical for this regulatory function (46). Additional factors may account for the superior efficacy observed with inhibition of tumor cell sialylation compared to Siglec blockade on macrophages: (i) Sialic acid reduction might decrease negative charge and electrostatic repulsion, which otherwise acts as a barrier to essential interactions that initiate phagocytosis (27), although this has not been thoroughly examined for antibody-mediated phagocytosis. (ii) Since macrophages in this study were derived from monocytes of healthy donors, Siglecs might play a less prominent role than they would with tumor-infiltrating immune cells. (iii) Protein sialylation could alter interactions between cancer cell proteins and macrophage activating receptors. (iv) Underlying glycan structures like galactose or GalNAc, which may be capped by sialic acid, could serve as immune modulators when exposed as terminal glycan monosaccharides.

Nevertheless, there is evidence that sialic acid/Siglec interactions also regulate immune escape *in vivo*. For example, sialyltransferase inhibition was effective in reducing tumor cell loads in xenografted models using SCID or CD89 transgenic SCID mice treated with anti-EGFR IgG1, -IgG2 or -IgA2 antibodies (29, 47). Indeed, Siglec-E, the homolog of human Siglec-7 and -9, is also expressed by infiltrating myeloid cells of different cancer types (28). For instance, desialylation of breast cancer cells in a syngeneic mouse model prolonged survival of mice through the engagement of Siglec-E on tumor-infiltrating myeloid cells (48). Blocking Siglec-7 and Siglec-9 by antibodies on myeloid cells in human Siglec-7/-9 transgenic mice confirmed the therapeutic potential of Siglec blocking antibodies for tumor immunotherapy (28). An additional promising approach consists of a HER2 antibody that has been conjugated to a sialidase to achieve sialic acid depletion specifically at the tumor site (48). The conjugate showed improved antitumor immune responses in mice through the engagement of Siglec-E on tumor infiltrating myeloid cells (48). In contrast to other studies (27, 49), we observed increased macrophage phagocytosis of tumor cells only in the presence of tumor targeting antibodies. In a subcutaneous melanoma mouse model, a gp75 tumor-targeting antibody successfully prevented tumor lesions in Siglec-E knockout (KO) mice compared to wild-type (WT) mice. However, no difference was observed between Siglec-E KO and WT mice when an isotype control antibody was used. This aligns with reports that the anti-tumor activity of Siglecs depends on an active Fc-FcR interaction, similar to what is required for CD47 blockade (28, 50).

In conclusion, the role of myeloid cells in the tumor microenvironment offers both challenges and opportunities for cancer immunotherapy. While often associated with immunosuppressive functions and poor prognosis, myeloid cells have also been identified as critical effector cells for antibody-based therapies. This study emphasizes that inhibition of sialylation may represent a more promising strategy to enhance ADCP by therapeutic antibodies compared to Siglec blockade. However, for therapeutic applications further developments are required to achieve sialylation inhibition specifically in tumor cells. Then, interference with tumor cell expressed sialic acid may become an effective approach to combine with monoclonal antibodies to fully harness the potential of myeloid cells in cancer immunotherapy and to overcome immune evasion employed by tumors.

## Data Availability

The original contributions presented in the study are included in the article/supplementary material. Further inquiries can be directed to the corresponding author/s.
